# 3-(2-Bromo­benz­yl)-1-methyl-1*H*-imidazol-3-ium bromide

**DOI:** 10.1107/S1600536811019842

**Published:** 2011-06-04

**Authors:** Jian-Yu Dong, Tian-Pa You

**Affiliations:** a Department of Chemistry, University of Science & Technology of China, Hefei, Anhui 230026, People’s Republic of China

## Abstract

In the title compound, C_11_H_12_BrN_2_^+^·Br^−^, the imidazole and phenyl rings are nearly perpendicular, making a dihedral angle of 87.71 (7)°. The crystal structure is stabilized by non-classical inter­molecular C—H⋯Br hydrogen bonds and inversion-related mol­ecules are linked through π–π inter­actions between the imidazole ring systems [centroid–centroid distance = 3.472 (6) Å].

## Related literature

Imidazolium salts are used to obtain transition metal complexes of *N*-heterocyclic carbenes, which have become an important class of catalysts in organometallic chemistry and organic synthesis, see: Marion & Nolan (2008 ▶); Herrmann (2002 ▶); Qin *et al.* (2006 ▶). For related structures, Guo *et al.* (2008 ▶); Liu *et al.* (2003 ▶).
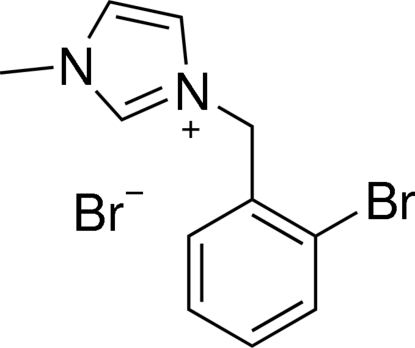


## Experimental

### 

#### Crystal data

C_11_H_12_BrN_2_^+^·Br^−^
*M* *_r_* = 332.05
Orthorhombic, 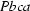

*a* = 8.4548 (10) Å
*b* = 13.9166 (13) Å
*c* = 20.831 (2) Å
*V* = 2451.1 (5) Å^3^
*Z* = 8
Mo *K*α radiation
μ = 6.58 mm^−1^
*T* = 298 K
0.42 × 0.40 × 0.21 mm

#### Data collection

Bruker SMART 1K CCD area-detector diffractometer
Absorption correction: multi-scan (*SADABS*; Sheldrick, 1996 ▶) *T*_min_ = 0.169, *T*_max_ = 0.339
9279 measured reflections
2158 independent reflections
1530 reflections with *I* > 2σ(*I*)
*R*_int_ = 0.126

#### Refinement

*R*[*F* ^2^ > 2σ(*F* ^2^)] = 0.064
*wR*(*F* ^2^) = 0.130
*S* = 1.19
2158 reflections
138 parameters
H-atom parameters constrained
Δρ_max_ = 0.74 e Å^−3^
Δρ_min_ = −0.65 e Å^−3^

### 

Data collection: *SMART* (Bruker, 2007 ▶); cell refinement: *SAINT* (Bruker, 2007 ▶); data reduction: *SAINT*; program(s) used to solve structure: *SHELXTL* (Sheldrick, 2008 ▶); program(s) used to refine structure: *SHELXTL*; molecular graphics: *SHELXTL*; software used to prepare material for publication: *SHELXTL*.

## Supplementary Material

Crystal structure: contains datablock(s) global, I. DOI: 10.1107/S1600536811019842/ff2011sup1.cif

Structure factors: contains datablock(s) I. DOI: 10.1107/S1600536811019842/ff2011Isup2.hkl

Supplementary material file. DOI: 10.1107/S1600536811019842/ff2011Isup3.cdx

Supplementary material file. DOI: 10.1107/S1600536811019842/ff2011Isup4.cml

Additional supplementary materials:  crystallographic information; 3D view; checkCIF report

## Figures and Tables

**Table 1:** Hydrogen-bond geometry (Å, °)

*D*—H⋯*A*	*D*—H	H⋯*A*	*D*⋯*A*	*D*—H⋯*A*
C4—H4*A*⋯Br2^i^	0.97	2.86	3.662 (6)	141

Symmetry code: (i) 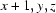.

**Fig. 1.**
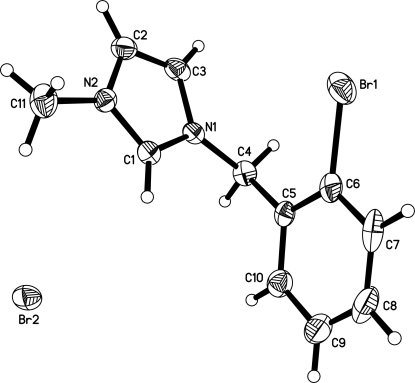
The molecular structure of the title compound, showing 30% probability displacement ellipsoids.

## supplementary crystallographic information

### Comment

Imidazolium salts or its derivatives are used as ionic liquids, and in many organic transformations. They are used to obtain transition metal complexes of N-heterocyclic carbenes which have become a very important class of catalysts in organometallic chemistry and organic synthesis (Herrmann, 2002, Marion & Nolan, 2008). We here report the crystal structure of the title compound.

Bond lengths and angles in the title molecule (Fig. 1) are within normal ranges. The imidazole and the phenyl ring are nearly perpendicular, with a dihedral angle of 87.71 (2)°.

The molecular structure is stabilized by C—H···Br hydrogen bonds (Table 1). The crystal structure is stabilized by π-π interactions between the imidazole ring systems of the inversion related molecules, with a *Cg*1···*Cg*1^i^ distance of 3.472 (6) Å [symmetry code: (i) 1-*x*, -*y*, 1-*z*].

### Experimental

1-methyl-1*H*-imidazole (0.615 g, 7.5 mmol) and 1-bromo-2-(bromomethyl)benzene (1.25 g, 5 mmol) in 20 ml of dioxane were refluxed for 12 h. After cooling the solution to room temperature, the mixture was filtered and afforded a colorless solid. Colourless single crystals suitable for X-ray diffraction were obtained by recrystallization from acetonitrile and diethyl ether.

### Refinement

H atoms were placed in calculated positions with C—H = 0.95–0.99 Å, and refined in riding mode with *U*_iso_(H) = 1.2–1.5*U*_eq_(C).

### Crystal data

**Table:**

C_11_H_12_BrN_2_^+^·Br^−^	*D*_x_ = 1.800 Mg m^−^^3^
*M**_r_* = 332.05	Mo *K*α radiation, λ = 0.71073 Å
Orthorhombic, *P**b**c**a*	Cell parameters from 992 reflections
*a* = 8.4548 (10) Å	θ = 2.6–25.2°
*b* = 13.9166 (13) Å	µ = 6.58 mm^−^^1^
*c* = 20.831 (2) Å	*T* = 298 K
*V* = 2451.1 (5) Å^3^	Block, colourless
*Z* = 8	0.42 × 0.40 × 0.21 mm
*F*(000) = 1296	

### Data collection

**Table:**

Bruker SMART 1K CCD area-detector diffractometer	2158 independent reflections
Radiation source: fine-focus sealed tube	1530 reflections with *I* > 2σ(*I*)
graphite	*R*_int_ = 0.126
φ and ω scans	θ_max_ = 25.0°, θ_min_ = 2.0°
Absorption correction: multi-scan (*SADABS*; Sheldrick, 1996)	*h* = −10→9
*T*_min_ = 0.169, *T*_max_ = 0.339	*k* = −15→16
9279 measured reflections	*l* = −15→24

### Refinement

**Table:**

Refinement on *F*^2^	Secondary atom site location: difference Fourier map
Least-squares matrix: full	Hydrogen site location: inferred from neighbouring sites
*R*[*F*^2^ > 2σ(*F*^2^)] = 0.064	H-atom parameters constrained
*wR*(*F*^2^) = 0.130	*w* = 1/[σ^2^(*F*_o_^2^) + (0.0439*P*)^2^ + 1.6882*P*] where *P* = (*F*_o_^2^ + 2*F*_c_^2^)/3
*S* = 1.19	(Δ/σ)_max_ < 0.001
2158 reflections	Δρ_max_ = 0.74 e Å^−^^3^
138 parameters	Δρ_min_ = −0.65 e Å^−^^3^
0 restraints	Extinction correction: *SHELXTL* (Sheldrick, 2008), Fc^*^=kFc[1+0.001xFc^2^λ^3^/sin(2θ)]^-1/4^
Primary atom site location: structure-invariant direct methods	Extinction coefficient: 0.0051 (4)

### Special details

**Table:**

Geometry. All e.s.d.'s (except the e.s.d. in the dihedral angle between two l.s. planes) are estimated using the full covariance matrix. The cell e.s.d.'s are taken into account individually in the estimation of e.s.d.'s in distances, angles and torsion angles; correlations between e.s.d.'s in cell parameters are only used when they are defined by crystal symmetry. An approximate (isotropic) treatment of cell e.s.d.'s is used for estimating e.s.d.'s involving l.s. planes.
Refinement. Refinement of *F*^2^ against ALL reflections. The weighted *R*-factor *wR* and goodness of fit *S* are based on *F*^2^, conventional *R*-factors *R* are based on *F*, with *F* set to zero for negative *F*^2^. The threshold expression of *F*^2^ > σ(*F*^2^) is used only for calculating *R*-factors(gt) *etc*. and is not relevant to the choice of reflections for refinement. *R*-factors based on *F*^2^ are statistically about twice as large as those based on *F*, and *R*- factors based on ALL data will be even larger.

### Fractional atomic coordinates and isotropic or equivalent isotropic displacement parameters (Å^2^)

**Table:**

	*x*	*y*	*z*	*U*_iso_*/*U*_eq_	
Br1	0.76580 (9)	0.05373 (6)	0.26368 (5)	0.0618 (4)	
Br2	0.09361 (7)	0.16571 (5)	0.46240 (4)	0.0464 (3)	
N1	0.6110 (5)	0.0692 (4)	0.4078 (3)	0.0320 (14)	
N2	0.4228 (6)	−0.0365 (4)	0.4189 (3)	0.0346 (14)	
C1	0.4546 (7)	0.0545 (5)	0.4031 (3)	0.0329 (17)	
H1	0.3803	0.1002	0.3908	0.039*	
C2	0.5601 (8)	−0.0806 (5)	0.4353 (4)	0.0444 (19)	
H2	0.5716	−0.1438	0.4490	0.053*	
C3	0.6759 (8)	−0.0158 (5)	0.4282 (4)	0.0434 (19)	
H3	0.7827	−0.0267	0.4359	0.052*	
C4	0.6935 (7)	0.1600 (5)	0.3952 (4)	0.0393 (19)	
H4A	0.8059	0.1473	0.3913	0.047*	
H4B	0.6785	0.2026	0.4315	0.047*	
C5	0.6377 (6)	0.2087 (5)	0.3363 (4)	0.0337 (18)	
C6	0.6590 (7)	0.1733 (5)	0.2750 (4)	0.041 (2)	
C7	0.6039 (9)	0.2206 (8)	0.2210 (4)	0.061 (2)	
H7	0.6185	0.1945	0.1803	0.073*	
C8	0.5265 (10)	0.3076 (8)	0.2288 (6)	0.069 (3)	
H8	0.4887	0.3404	0.1930	0.083*	
C9	0.5056 (9)	0.3454 (7)	0.2885 (6)	0.068 (3)	
H9	0.4543	0.4040	0.2933	0.082*	
C10	0.5604 (8)	0.2968 (5)	0.3419 (4)	0.049 (2)	
H10	0.5454	0.3233	0.3824	0.059*	
C11	0.2652 (7)	−0.0794 (6)	0.4187 (4)	0.056 (2)	
H11A	0.1878	−0.0308	0.4279	0.084*	
H11B	0.2599	−0.1288	0.4508	0.084*	
H11C	0.2442	−0.1068	0.3773	0.084*	

### Atomic displacement parameters (Å^2^)

**Table:**

	*U*^11^	*U*^22^	*U*^33^	*U*^12^	*U*^13^	*U*^23^
Br1	0.0619 (5)	0.0619 (6)	0.0615 (8)	−0.0090 (4)	0.0169 (4)	−0.0205 (5)
Br2	0.0390 (4)	0.0502 (5)	0.0500 (7)	0.0086 (3)	−0.0099 (3)	0.0048 (4)
N1	0.030 (3)	0.041 (3)	0.025 (4)	0.007 (3)	−0.002 (2)	0.000 (3)
N2	0.041 (3)	0.035 (3)	0.029 (4)	−0.001 (3)	0.002 (3)	0.000 (3)
C1	0.031 (3)	0.045 (4)	0.023 (5)	0.006 (3)	−0.005 (3)	0.002 (3)
C2	0.049 (4)	0.042 (4)	0.043 (6)	0.010 (4)	0.003 (3)	0.002 (4)
C3	0.041 (4)	0.056 (5)	0.034 (5)	0.020 (4)	−0.003 (3)	0.003 (4)
C4	0.034 (3)	0.045 (4)	0.039 (6)	−0.005 (3)	−0.005 (3)	−0.003 (4)
C5	0.025 (3)	0.039 (4)	0.037 (6)	−0.011 (3)	−0.002 (3)	0.004 (4)
C6	0.032 (3)	0.044 (4)	0.048 (6)	−0.019 (3)	0.004 (3)	−0.002 (4)
C7	0.055 (5)	0.094 (7)	0.033 (6)	−0.031 (5)	0.003 (4)	0.013 (5)
C8	0.051 (5)	0.087 (8)	0.069 (9)	−0.010 (5)	−0.003 (5)	0.045 (6)
C9	0.057 (5)	0.066 (6)	0.081 (9)	0.003 (4)	0.010 (5)	0.030 (6)
C10	0.045 (4)	0.051 (5)	0.052 (7)	−0.005 (3)	0.000 (4)	0.005 (4)
C11	0.045 (4)	0.062 (5)	0.060 (7)	−0.015 (4)	0.001 (4)	−0.005 (5)

### Geometric parameters (Å, °)

**Table:**

Br1—C6	1.908 (7)	C5—C6	1.380 (10)
N1—C1	1.342 (7)	C5—C10	1.393 (9)
N1—C3	1.372 (8)	C6—C7	1.385 (11)
N1—C4	1.466 (8)	C7—C8	1.386 (13)
N2—C1	1.336 (9)	C7—H7	0.9300
N2—C2	1.357 (8)	C8—C9	1.362 (14)
N2—C11	1.460 (8)	C8—H8	0.9300
C1—H1	0.9300	C9—C10	1.382 (12)
C2—C3	1.338 (10)	C9—H9	0.9300
C2—H2	0.9300	C10—H10	0.9300
C3—H3	0.9300	C11—H11A	0.9600
C4—C5	1.480 (10)	C11—H11B	0.9600
C4—H4A	0.9700	C11—H11C	0.9600
C4—H4B	0.9700		
			
C1—N1—C3	106.5 (6)	C10—C5—C4	118.9 (7)
C1—N1—C4	126.0 (5)	C5—C6—C7	122.6 (8)
C3—N1—C4	127.5 (5)	C5—C6—Br1	119.2 (6)
C1—N2—C2	108.6 (5)	C7—C6—Br1	118.2 (7)
C1—N2—C11	124.8 (6)	C6—C7—C8	118.6 (9)
C2—N2—C11	126.6 (6)	C6—C7—H7	120.7
N2—C1—N1	109.0 (5)	C8—C7—H7	120.7
N2—C1—H1	125.5	C9—C8—C7	120.4 (9)
N1—C1—H1	125.5	C9—C8—H8	119.8
C3—C2—N2	107.1 (6)	C7—C8—H8	119.8
C3—C2—H2	126.5	C8—C9—C10	120.2 (9)
N2—C2—H2	126.5	C8—C9—H9	119.9
C2—C3—N1	108.8 (6)	C10—C9—H9	119.9
C2—C3—H3	125.6	C9—C10—C5	121.3 (9)
N1—C3—H3	125.6	C9—C10—H10	119.3
N1—C4—C5	113.1 (5)	C5—C10—H10	119.3
N1—C4—H4A	109.0	N2—C11—H11A	109.5
C5—C4—H4A	109.0	N2—C11—H11B	109.5
N1—C4—H4B	109.0	H11A—C11—H11B	109.5
C5—C4—H4B	109.0	N2—C11—H11C	109.5
H4A—C4—H4B	107.8	H11A—C11—H11C	109.5
C6—C5—C10	116.9 (7)	H11B—C11—H11C	109.5
C6—C5—C4	124.2 (7)		
			
C2—N2—C1—N1	1.3 (8)	N1—C4—C5—C10	114.2 (7)
C11—N2—C1—N1	−179.2 (7)	C10—C5—C6—C7	−1.5 (9)
C3—N1—C1—N2	−0.9 (8)	C4—C5—C6—C7	179.4 (6)
C4—N1—C1—N2	−179.3 (6)	C10—C5—C6—Br1	179.4 (4)
C1—N2—C2—C3	−1.1 (8)	C4—C5—C6—Br1	0.3 (8)
C11—N2—C2—C3	179.4 (7)	C5—C6—C7—C8	1.0 (10)
N2—C2—C3—N1	0.5 (9)	Br1—C6—C7—C8	−179.8 (5)
C1—N1—C3—C2	0.3 (8)	C6—C7—C8—C9	0.0 (11)
C4—N1—C3—C2	178.6 (7)	C7—C8—C9—C10	−0.5 (12)
C1—N1—C4—C5	−44.1 (9)	C8—C9—C10—C5	0.0 (11)
C3—N1—C4—C5	137.9 (7)	C6—C5—C10—C9	1.0 (10)
N1—C4—C5—C6	−66.7 (8)	C4—C5—C10—C9	−179.9 (6)

### Hydrogen-bond geometry (Å, °)

**Table:**

*D*—H···*A*	*D*—H	H···*A*	*D*···*A*	*D*—H···*A*
C4—H4A···Br2^i^	0.97	2.86	3.662 (6)	141

Symmetry codes: (i) *x*+1, *y*, *z*.
